# Enhanced immune complex formation in the lungs of patients with dermatomyositis

**DOI:** 10.1186/s12931-023-02362-0

**Published:** 2023-03-19

**Authors:** Yoshiaki Zaizen, Masaki Okamoto, Koichi Azuma, Junya Fukuoka, Hironao Hozumi, Noriho Sakamoto, Takafumi Suda, Hiroshi Mukae, Tomoaki Hoshino

**Affiliations:** 1 Division of Respirology, Neurology, and Rheumatology, Department of Medicine, Kurume University School of Medicine, 67 Asahi-Machi, Kurume, Fukuoka 830-0011 Japan; 2 Department of Pathology, Nagasaki University Graduate School of Biomedical Sciences, 1-7-1 Sakamoto, Nagasaki, 852-8501 Japan; 3 Department of Respirology and Clinical Research Center, National Hospital Organization Kyushu Medical Center, 1-8-1 Jigyouhama, Chuo-Ku, Fukuoka, 810-8563 Japan; 4 Second Division, Department of Internal Medicine, Hamamatsu University School of Medicine, 1-20-1 Handayama, Higashi-Ku, Hamamatsu, Sizuoka 431-3192 Japan; 5 Department of Respiratory Medicine, Nagasaki University Graduate School of Biomedical Sciences, 1-7-1 Sakamoto, Nagasaki, 82-8501 Japan; 6 Cancer Innovation Laboratory (CIL), Center for Cancer Research (CCR), National Cancer Institute (NCI)-Frederick, 1050 Boyles St, MD 21702-1201 Frederick, USA

**Keywords:** Acute exacerbation, Anti-MDA5 antibody, Dermatomyositis, Idiopathic pulmonary fibrosis, Immune complex, Immunoglobulin, Interstitial pneumonia, Rapidly progressive interstitial lung disease, Type III hypersensitivity

## Abstract

**Background:**

Interstitial lung disease is frequently comorbid with dermatomyositis and has a poor prognosis, especially in patients with the anti-melanoma differentiation-associated gene 5 (MDA5) autoantibody. However, the pathogenesis of dermatomyositis-related interstitial lung disease remains unclear.

**Methods:**

We examined 18 and 19 patients with dermatomyositis-related interstitial lung disease and idiopathic pulmonary fibrosis (control), respectively. Lung tissues obtained from these patients were semi-quantitatively evaluated by immunohistochemical staining with in-house anti-human MDA5 monoclonal antibodies, as well as anti-human immunoglobulin (Ig) G, IgM, IgA, and complement component 3(C3) antibodies. We established human MDA5 transgenic mice and treated them with rabbit anti-human MDA5 polyclonal antibodies, and evaluated lung injury and Ig and C3 expression.

**Results:**

MDA5 was moderately or strongly expressed in the lungs of patients in both groups, with no significant differences between the groups. However, patients with dermatomyositis-related interstitial lung disease showed significantly stronger expression of C3 (p < 0.001), IgG (p < 0.001), and IgM (p = 0.001) in the lungs than control. Moreover, lung C3, but IgG, IgA, nor IgM expression was significantly stronger in MDA5 autoantibody-positive dermatomyositis-related interstitial lung disease (n = 9) than in MDA5 autoantibody-negative dermatomyositis-related interstitial lung disease (n = 9; p = 0.022). Treatment with anti-MDA5 antibodies induced lung injury in MDA5 transgenic mice, and strong immunoglobulin and C3 expression was observed in the lungs of the mice.

**Conclusion:**

Strong immunoglobulin and C3 expression in the lungs involve lung injury related to dermatomyositis-related interstitial lung disease. Enhanced immune complex formation in the lungs may contribute to the poor prognosis of MDA5 autoantibody-positive dermatomyositis-related interstitial lung disease.

**Supplementary Information:**

The online version contains supplementary material available at 10.1186/s12931-023-02362-0.

## Introduction

Idiopathic inflammatory myopathies (IIMs) are rare autoimmune diseases [1]. Dermatomyositis is an IIM subtype presenting with proximal skeletal muscle weakness and muscle inflammation. Dermatomyositis is characterized by skin disorders, including Gottron’s sign and the heliotrope sign, with some patients showing definite cutaneous manifestations of dermatomyositis along with slight or clinically non-significant myopathy, which is defined as clinically amyopathic dermatomyositis (CADM) [2].

Interstitial lung disease (ILD) is relatively common among dermatomyositis patients and affects their prognosis. Specifically, patients with CADM are at a high risk of developing rapidly progressive interstitial lung disease (RP-ILD) with a poor prognosis due to resistance to immunosuppressive therapy [1]. Although various autoantibodies are involved in dermatomyositis [3, 4], the detailed mechanism underlying the immune response in dermatomyositis-related ILD (DM-ILD) remains unclear.

The anti-melanoma differentiation-associated gene 5 (MDA5) autoantibody [5] is among the main autoantibodies in DM-ILD. Anti-MDA5 autoantibodies have been often detected in high titers in patients with CADM and can be used as a biomarker in the clinical diagnosis of dermatomyositis; moreover, the presence of anti-MDA5 autoantibodies is associated with a poor survival prognosis, especially in DM-ILD patients [6, 7]. We have previously shown that low serum titers of anti-MDA5 autoantibody (< 100.0 IU/mL) or the absence of autoantibodies improved the survival rate of patients with DM-ILD and RP-ILD [8]. However, the role of anti-MDA5 autoantibodies in respiratory failure development in patients with DM-ILD remains unclear.

Several studies have reported a relationship between autoimmune disease and MDA5 [9]. A genome-wide association study revealed a significant association of single-nucleotide polymorphisms of MDA5 with resistance to type 1 diabetes [10] and the Aicardi–Goutières syndrome [11]. Gateva et al. reported that human MDA5 single-nucleotide polymorphisms (A946T) are risk variants for systemic lupus erythematosus (SLE) [12]. Robinson et al. reported that this single-nucleotide polymorphism was a gain-of-function mutation in SLE [13]. In particular, SLE is a leading cause of the third type of hypersensitivity in the Gell–Coombs classification (type III hypersensitivity), which causes deposition of immune complexes including complement and immunoglobulins in tissues throughout the body. Such type III allergic reactions may be closely related to dysfunction of MDA5.

We hypothesized that the third type of hypersensitivity in the Gell–Coombs classification (type III hypersensitivity) [14–16] is present in the lungs of patients with DM-ILD, especially in patients with anti-MDA5 autoantibodies. This study aimed to investigate the expression of immunoglobulins (IgG, IgM, IgA) and complement component 3 (C3) in the lungs of patients with DM-ILD and those with idiopathic pulmonary fibrosis (IPF) as control. We also have successfully produced in-house anti-human MDA5 polyclonal and monoclonal antibodies and used these antibodies to examine the expression of MDA5 protein in the lungs of DM-ILD and IPF patients. Using the human surfactant promoter SPC, we also established transgenic mice overexpressing full-length human MDA5 protein in the lungs.

## Methods

### Study participants

This retrospective study included 18 patients diagnosed with DM-ILD at our institutions between 1997 and 2020. The patients met the diagnostic criteria for polymyositis and dermatomyositis as reported by Bohan and Peter [17, 18] or the diagnostic criteria for CADM reported by Sontheimer [2], while patients met the diagnostic criteria of RP-ILD described by Kondoh et al. [19] Additionally, we investigated 19 patients, as control, histologically diagnosed with IPF, including nine patients showing acute IPF exacerbation. All patients were diagnosed as having IPF and/or IPF-AE by multidisciplinary discussion based on the official global guidelines for IPF [20, 21]. The sample size of this comparative group was determined using Student’s *t*-test with a detection power and significance level of 80% and 5%, respectively, based on the results of a preliminary study. We examined a single section of lung specimens showing obvious lung injury and obtained through surgical lung biopsy (SLB), lung transplantation, or autopsy. In addition, specimen paraffin blocks for each case were retrieved from the archives of the participating institutions. We also collected the patients’ background characteristics; information regarding the applied diagnostic criteria of IIMs; and anti-MDA5 antibody test results. Additionally, as controls for immunohistochemistry staining, we investigated two cases without any underlying respiratory disease that involved lung resection for lung cancer.

### Establishment of an anti-human MDA5 polyclonal and monoclonal antibodies

Full-length human MDA5 cDNA (GenBank Accession No. AF095844) with a 6X His tag, GST, and turbo 3C protease cleavage site of Leu-Glu-Val-Leu-Phe-Gln-Gly-Pro at the N-terminus was subcloned into the pPSC8 expression vector (Protein Sciences Corporation, Meriden, CT, USA); subsequently designated as pPSC8/human MDA5. Recombinant human MDA5 protein was isolated from SF9 cells co-transfected with baculovirus AcNPV and pPSC8/human MDA5. To generate antiserum, specific pathogen-free (Japanese White) rabbits were immunized with recombinant human MDA5 protein Purified antibody was generated from the antisera using a protein G column (Cytiva, Tokyo, Japan), as reported previously [22, 23]. An anti-human MDA5 monoclonal antibody (mAb) clone H27 (mouse IgG1) and H46 (mouse IgG2b) was established by fusing mouse myeloma cell line X-63·Ag8/653 with spleen cells isolated from a BALB/c mouse immunized with the recombinant human MDA5 protein, as reported previously [22, 23].

### Generation of transgenic (Tg) mice constitutively overproducing human MDA5 in the lung and a mouse model of lung injury caused by anti-human MDA5 antibody

Male and female C57BL/6N (B6) and B6D2F1 (BDF1) mice were purchased from Charles River Japan (Yokohama, Japan) and bred in our laboratory. Transgenic (Tg) mice were generated as described previously [24]. Briefly, full-length human MDA5 cDNA (GenBank Accession No. AF095844) was generated and subcloned into the SalI site of a 3.7SPC/SV40 vector containing the SP-C promoter, the SV40 small T intron, and a polyA signal (kindly provided by Dr. Jeffrey A. Whitsett, Cincinnati Children’s Hospital Medical Center, OH), and was designated as SPC-MDA5. The NdeI- and NotI-digested linear DNA fragments were injected into fertilized eggs of BDF1 mice. Hemizygous Tg mice were generated by mating founder mice with B6 mice. We generated three lines of the human MDA5 transgenic mice (line no. 32, 55, and 116. In this study, we mainly used line no. 55 of human MDA5 transgenic mice. The lung injury model was established by treating human MDA5 transgenic mice and wild-type BDF1 mice (n = 4 to 7 per group) with 0.5 mL of rabbit anti-human MDA5 polyclonal antibodies (antisera) or normal rabbit sera 4 times (at day 0, 7, 14, 21) or eight times (at day 0, 7, 14, 21, 28, 35, 42, 49). Mice were sacrificed at day 28 or 56 for histological examination. Three repeated experiments were performed. Human MDA5 transgenic mice and wild-type BDF1 mice were also treated with 1 mg of purified rabbit anti-human MDA5 polyclonal antibodies or 1 mg of rabbit IgG (Sigma-Aldrich, Tokyo, Japan).

### Immunohistochemical staining and RNA in situ hybridization

Immunohistochemistry staining in human specimens was performed as reported previously [25]. Briefly, tissues were fixed with 10% buffered formalin and embedded in paraffin wax. Serial sections (4-μm-thick) were cut from the paraffin-embedded tissues and placed on poly-l-lysine-coated slides. Deparaffinized sections were autoclaved for 3 min in 10 mM citric acid buffer (pH 6.0). The sections were incubated in 0.3% H_2_O_2_ for 10 min to block endogenous peroxidase activity; subsequently, they were stained using mouse anti-human MDA5 mAb (H27; 1.2 μg/mL), rabbit anti-human IgG (P0214; Agilent, Palo Alto, CA, USA; × 100), rabbit anti-human IgM (P0215; Agilent; × 50), rabbit anti-human IgA (P0216; Agilent; × 100), and rabbit anti-human complement C3c (A0062, Agilent; × 5000). Mouse IgG1 (BioLegend, Tokyo) was used as the control. The sections were treated with these antibodies at room temperature for 2 h. Positive reactivity was identified using goat anti-mouse and anti-rabbit immunoglobulins (Ig) conjugated to peroxidase-labeled polymer (EnVision Dual Link system-HRP, Agilent) and Liquid DAB + Substrate Chromogen System Liquid (Agilent).

We also investigated the expression of human MDA5, Ig, and complement protein in human MDA5 transgenic mice and its lung injury model by immunohistochemistry. Immunohistochemistry staining was performed for Ig and complement protein utilizing standard methods with the following antibodies: anti-mouse IgG rabbit polyclonal antibody (A90-117A; Bethyl Laboratories, Montgomery, TX, USA; × 2000), anti-mouse IgA rabbit polyclonal antibody (A90-104A; Bethyl Laboratories; × 2000), anti-mouse IgM rabbit polyclonal antibody (A90-102A; Bethyl Laboratories; × 2000), and anti-C3 rabbit monoclonal antibody clone (EPR19394; Abcam, Cambridge, UK; × 2000). Immunohistochemistry staining of anti-human MDA5 mouse mAb was performed as follows. Deparaffinized sections were autoclaved for 10 min in 10 mM citric acid buffer (pH 6.0). The sections were incubated in 0.3% H_2_O_2_ for 15 min to block endogenous peroxidase activity; subsequently, they were stained with anti-human MDA5 mouse mAb (H27 and H46; 10 µg/mL). The sections were treated with these antibodies at room temperature overnight. Positive reactivity was identified with a labeled streptavidin biotinylated antibody 2 kit (K0675; Agilent).

**Fig. 1:**
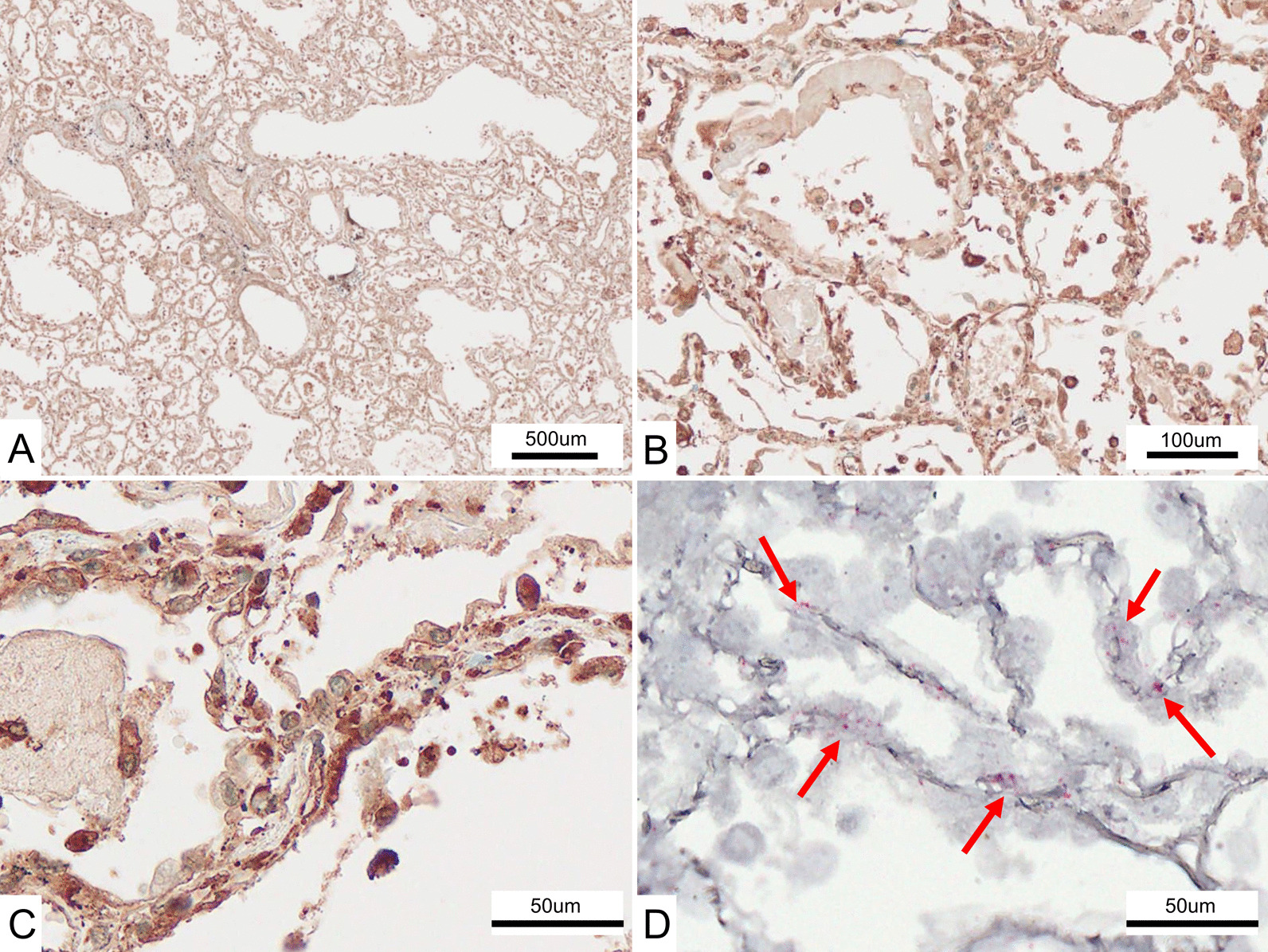
Comparative analysis of melanoma differentiation-associated gene 5 (MDA5) RNA in situ hybridization and immunohistochemical (IHC) staining using anti-human MDA5 monoclonal antibody (mAb) (clone H27) in a patient with dermatomyositis-related interstitial lung disease (DM-ILD). **A** Low-power view of IHC staining using the anti-MDA5 mAb clone H27. **B** Mid-power view. **C** High-power view. Damaged and foamy pneumocytes, alveolar macrophages, and fibrin were strongly positive. **D** High-power view of MDA5 RNA in situ hybridization. Damaged pneumocytes were positive (red arrows)

To evaluate the efficacy of two anti-human MDA5 monoclonal antibodies (clone H27 and H46; 3.0 μg/mL), we performed ribonucleic acid (RNA) in situ hybridization in lung samples from two patients with DM-ILD. We analyzed human MDA5 messenger RNA (mRNA) in formalin-fixed paraffin-embedded samples using an RNA in situ hybridization kit (RNAscope^®^ 2.5HD Reagent Kit-RED; Advanced Cell Diagnostics, CA, USA), as reported previously [25]. Positive signals for fast red were analyzed using a fluorescent microscope (KEYENCE, Osaka, Japan). We compared slides showing immunohistochemistry staining with mouse anti-human MDA5 mAbs and in situ hybridization slides. We also confirmed that both slides were equally positive for injured alveolar epithelium (Fig. 1). On the basis of these results, we evaluated MDA5 expression in the alveolar epithelium by immunohistochemistry staining and used the in-house anti-human MDA5 mAb clone H27 for evaluation via immunohistochemistry staining.

### Pathological assessment

Two pathologists (YZ and JF) who were blinded with clinical data performed semi-quantitative immunohistochemistry scoring in human patient samples. They independently examined the pathological slides, recorded their impressions in a blinded manner, and discussed their evaluation of severity based on immunohistochemistry staining in each case. We assessed the immunohistochemistry staining intensity in the alveolar epithelium, but not in the fibrin or macrophages within the alveoli. Slides were rated based as follows: score 0, negative as in controls; score 1, weakly positive; score 2, moderately positive; and score 3, strongly positive. The histological photographs showing the scores for each immunohistochemistry staining were presented in Additional file 1: Fig. S1 and Additional file 1: Fig. S2. In addition, we investigated the expression locations of MDA5 in human MDA5 transgenic mice and of Ig and complement proteins in a model of lung injury using anti-human MDA5 polyclonal antibodies by immunohistochemistry.

### Statistical analysis

Numerical data for patients’ characteristics are presented as median values with a 25–75% interquartile range; between-group comparisons were performed using the Mann–Whitney *U* test or Fisher’s exact test, as appropriate. Data for semi-quantitative immunohistochemistry scoring are presented as means ± standard deviations, with between-group comparisons using the Mann–Whitney *U* test. Statistical significance was set at p < 0.05. All analyses were performed using JMP software (version 14.0; SAS Institute, Cary, NC, USA).

### Ethical issues

This study was conducted in accordance with the tenets of the Declaration of Helsinki and approved by the Institutional Review Board of our institute (approval date, July 31, 2019, No. 19090). All procedures were approved by the Committee on the Ethics of Animal Experiments, Kurume University (Approval No. 2022-083, 2022-084, 2022-085). Animal care was provided in accordance with the procedures outlined in the “Principles of laboratory animal care” (National Institutes of Health Publication 86-23, revised 1985). All efforts were made to minimize the suffering of animals used in this study.

## Results

### Patient characteristics

Table 1 presents the patient characteristics. We evaluated 18 and 19 patients showing DM-ILD and IPF, respectively. The DM-ILD group included younger patients (p = 0.001) and more females (p = 0.001) than the IPF group. In the DM-ILD group (n = 18), six, one, and eleven cases involved autopsy, lung transplant, and SLB, respectively. Nine (50%) of the 18 DM-ILD cases showed seropositivity for the anti-MDA5 antibody, while six and three cases involved autopsy and SLB, respectively. Thus, all six autopsy cases showed seropositivity for anti-MDA5 antibodies. Seven of the nine anti-MDA5 antibody-seropositive DM-ILD cases showed a clinical course of RP-ILD. Moreover, the lung tissues of all nine seropositive DM-ILD cases showed a diffuse alveolar damage (DAD) pattern. Among the nine cases of anti-MDA5 antibody-seronegative DM-ILD were positive for anti-Aminoacyl-tRNA Synthetase antibodies. Additionally, two, one, and six cases showed DAD, usual interstitial pneumonia (UIP), or nonspecific interstitial pneumonia (NSIP) patterns, respectively.

**Table 1:** Patient characteristics

	DM-ILD group	IPF group	P value
Number	18	19	
Age	54 (46–63)	65 (62–67)	0.001
Sex: male	6 (33%)	18 (95%)	0.001
RP-ILD or IPF-AE	9 (50%)	9 (47%)	
Tissue collection method			
SLB	11 (61%)	10 (53%)	
Transplant	1 (6%)	0 (0%)	
Autopsy	6 (33%)	9 (47%)	
Dx of IIMs			
DM	12 (67%)	–	
CADM	6 (33%)	–	
Histological pattern			
DAD	11 (61%)	9 (47%)	
NSIP	6 (33%)	0 (0%)	
UIP	1 (6%)	10 (53%)	
Treatment for autopsy and lung transplant cases			
Corticosteroid	7 (100%)	8 (89%)	
Calcineurin inhibitors	6 (86%)	2 (22%)	
Cyclophosphamide	5 (71%)	3 (33%)	
Cause of death in autopsy cases			
Respiratory failure (including IPF-AE or RP-ILD)	6 (100%)	6 (67%)	
Lung cancer	0 (0%)	3 (33%)	

*AE* acute exacerbation; *CADM* clinical amyopathic dermatomyositis *DAD* diffuse alveolar damage; *DM* dermatomyositis; *Dx* diagnosis; *IIMs* idiopathic inflammatory myopathies; *ILD* interstitial lung disease; *IPF* idiopathic pulmonary fibrosis; *NSIP* nonspecific interstitial pneumonia; *RP-ILD* rapidly progressive interstitial lung disease; *SLB* surgical lung biopsy; *UIP* usual interstitial pneumonia

In the IPF group (control, n = 19), nine (47%) patients experienced acute exacerbations (AEs) and were histopathologically diagnosed as showing DAD patterns at autopsy. The remaining 10 (53%) patients showed a chronic course and were diagnosed as showing a UIP pattern through SLB.

All seven cases (100%, six autopsies and one lung transplant) and eight of nine cases (89%) in the DM-ILD and IPF groups, respectively, were treated with corticosteroids. Additionally, calcineurin inhibitors and cyclophosphamide were used in six and five DM-ILD cases and in two and three IPF autopsies, respectively (Table 1). Tests for significance for these differences were not calculated as the usage of steroids and immunosuppressive agents in DM-ILD and IPF groups were different.

Among the 15 autopsies (6 DM-ILD and 9 IPF cases), 3 were of patients in the IPF group who died from lung cancer. The remaining 12 patients died due to progressive respiratory failure, including acute exacerbation of interstitial lung disease. Additionally, one patient in the DM-ILD group underwent lung transplantation because of progressive respiratory failure.

### MDA5 overexpression in lungs of patients with DM-ILD and IPF

**Fig. 2:**
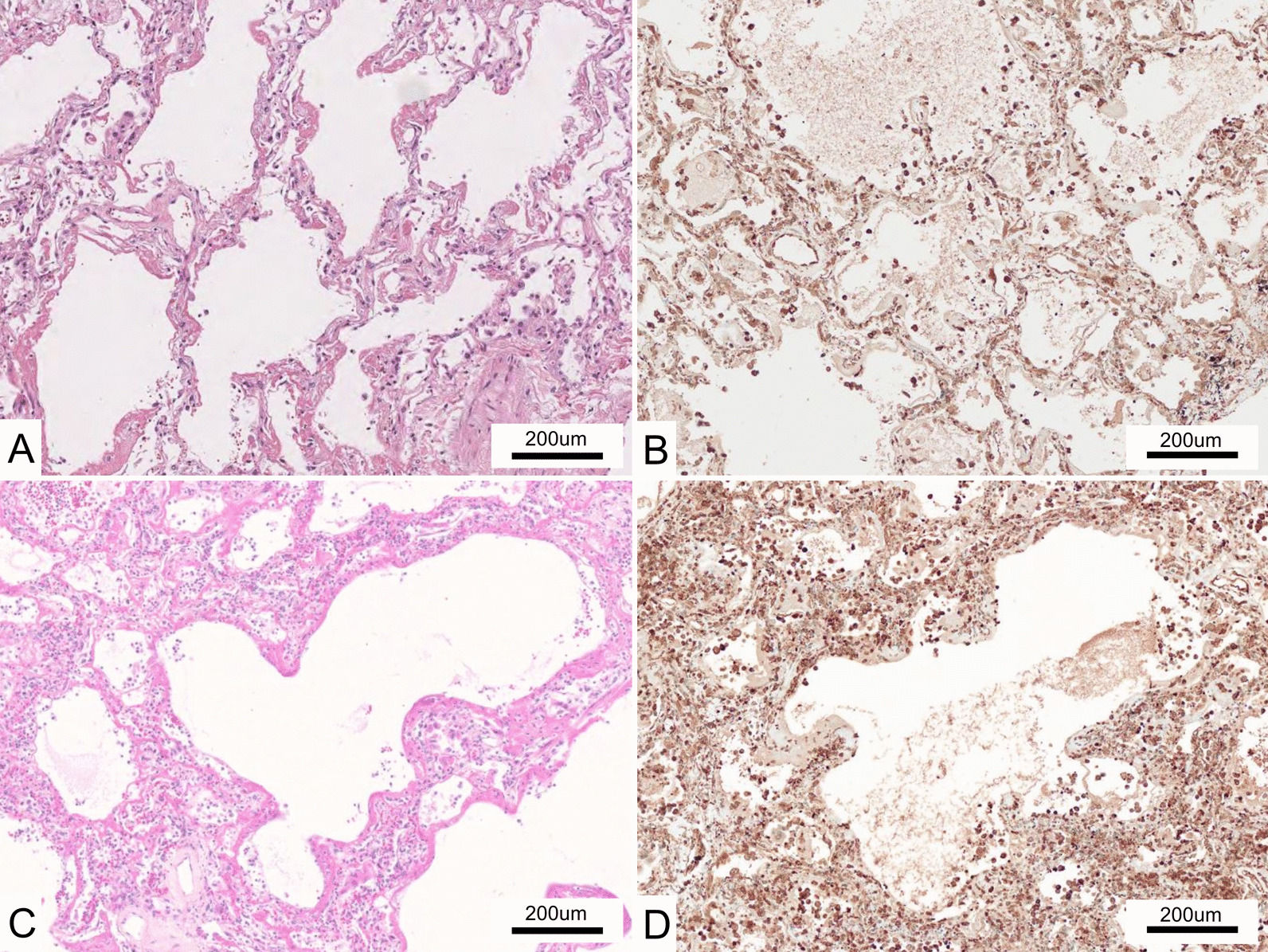
Histopathological findings in the lungs of patients with DM-ILD and idiopathic pulmonary fibrosis (IPF). **A** Hematoxylin & eosin (H&E) staining of a patient with DM-ILD. This patient showed an exudative diffuse alveolar damage (DAD) pattern. **B** IHC staining with anti-MDA5 monoclonal antibody (mAb) (clone H27) in a patient with DM-ILD. MDA5 is extensively and strongly expressed in the alveolar epithelium. **C** H&E staining in a patient with IPF. This patient showed an exudative DAD pattern, as well as a usual interstitial pneumonia (UIP) pattern in the background. **D** IHC staining with anti-MDA5 mAb (clone H27) in a patient with IPF. There was strong MDA expression in the alveolar epithelium

Immunohistochemistry analysis revealed moderate (n = 15) or strong (n = 2) MDA5 expression in the lungs of 17/18 DM-ILD patients and weak expression in one patient. No correlations were observed among the histopathological pattern, tissue collection method, and MDA5 expression. Notably, all 19 IPF cases showed moderate (n = 14) or strong (n = 5) MDA5 expression in the lungs. Strong MDA5 expression was observed in the lungs of both DM-ILD and IPF groups (Fig. 2b, d). However, no significant different between two group was observed in the expression intensity (p = 0.156) (Table 2).

**Table 2:** Evaluation of immunohistochemical staining

Expression intensity 0/1/2/3 (mean ± SD)	DM-ILD (n = 18)	IPF (n = 19)	P value
C3c	3/7/6/2 (1.389 ± 0.916)	17/2/0/0 (0.105 ± 0.315)	< 0.001
IgG	0/4/12/2 (1.889 ± 0.583)	4/10/5/0 (1.053 ± 0.705)	< 0.001
IgM	7/10/1/0 (0.667 ± 0.594)	17/2/0/0 (0.105 ± 0.315)	0.001
IgA	5/11/0/2 (0.944 ± 0.873)	4/9/6/0 (1.105 ± 0.737)	0.323
MDA5	0/1/15/2 (2.056 ± 0.416)	0/0/14/5 (2.263 ± 0.452)	0.161

Expression intensity score 0, negative staining; score 1, weakly positive; score 2, moderately positive; and score 3, strongly positive. Details are described in the “Methods” section

*DM* dermatomyositis; *ILD* interstitial lung disease; *IPF* idiopathic pulmonary fibrosis; *MDA5* melanoma differentiation-associated gene 5

### Strong expression of complement and ig in the lungs of DM-ILD but not IPF patients

**Fig. 3:**
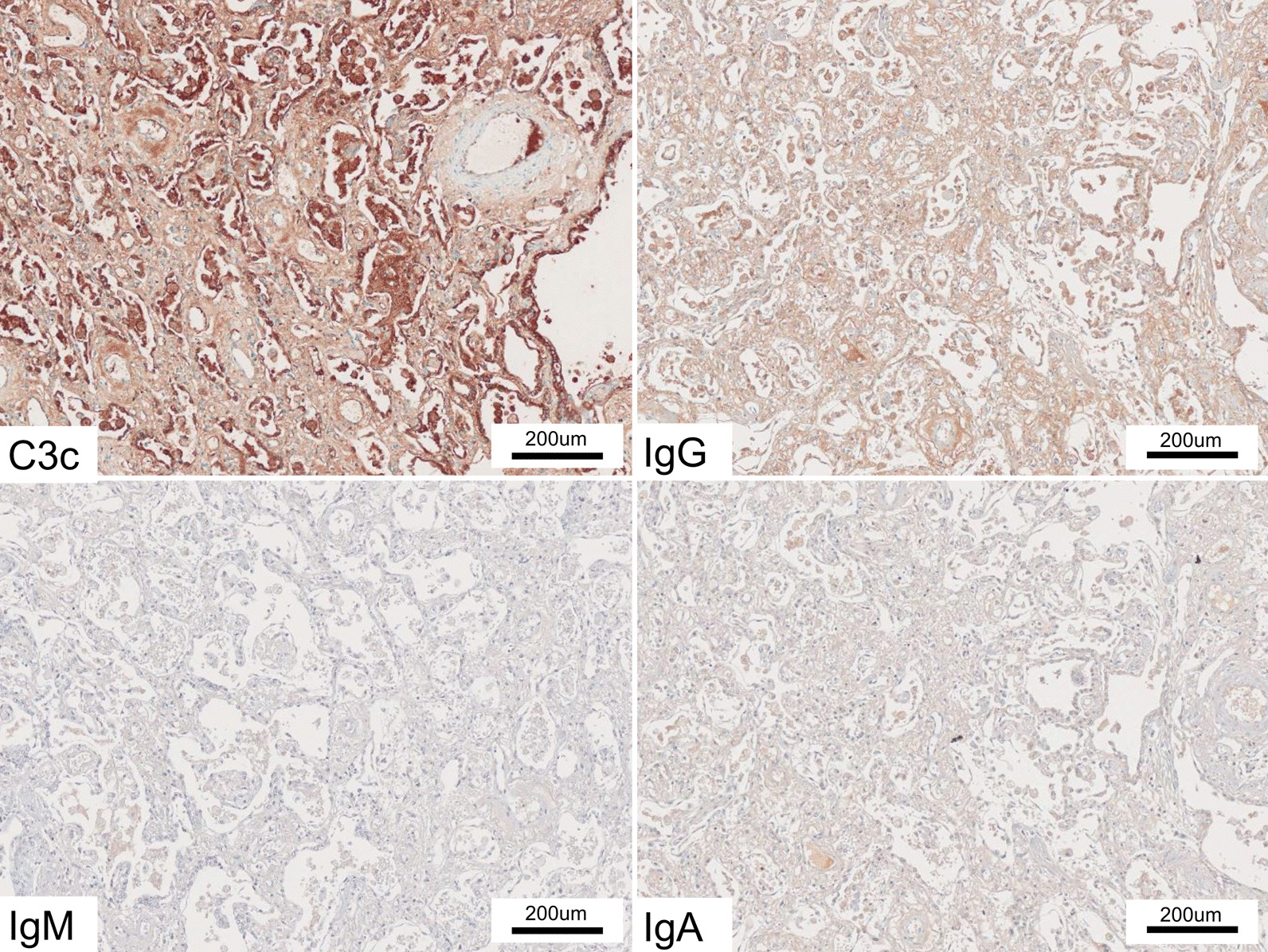
IHC staining with complement protein and immunoglobulins (Ig) in the lungs of a patient with DM-ILD. This case shows strong expression of complement C3c and IgG, as well as weak expression of IgM and IgA, in alveolar cells

Figure 3 shows strong expression of complement C3c and IgG in the lungs of DM-ILD patients. Immunohistochemistry staining for complement C3c showed positive results in 15/18 cases, of which eight cases showed moderate or greater intensity. All 18 cases showed positive results for IgG, while 11 cases showed positive results for IgM. Interestingly, 13/18 cases showed positive results for IgA; however, only two cases showed strongly positive results, while 11 cases showed weakly positive results (Table 2).

**Fig. 4:**
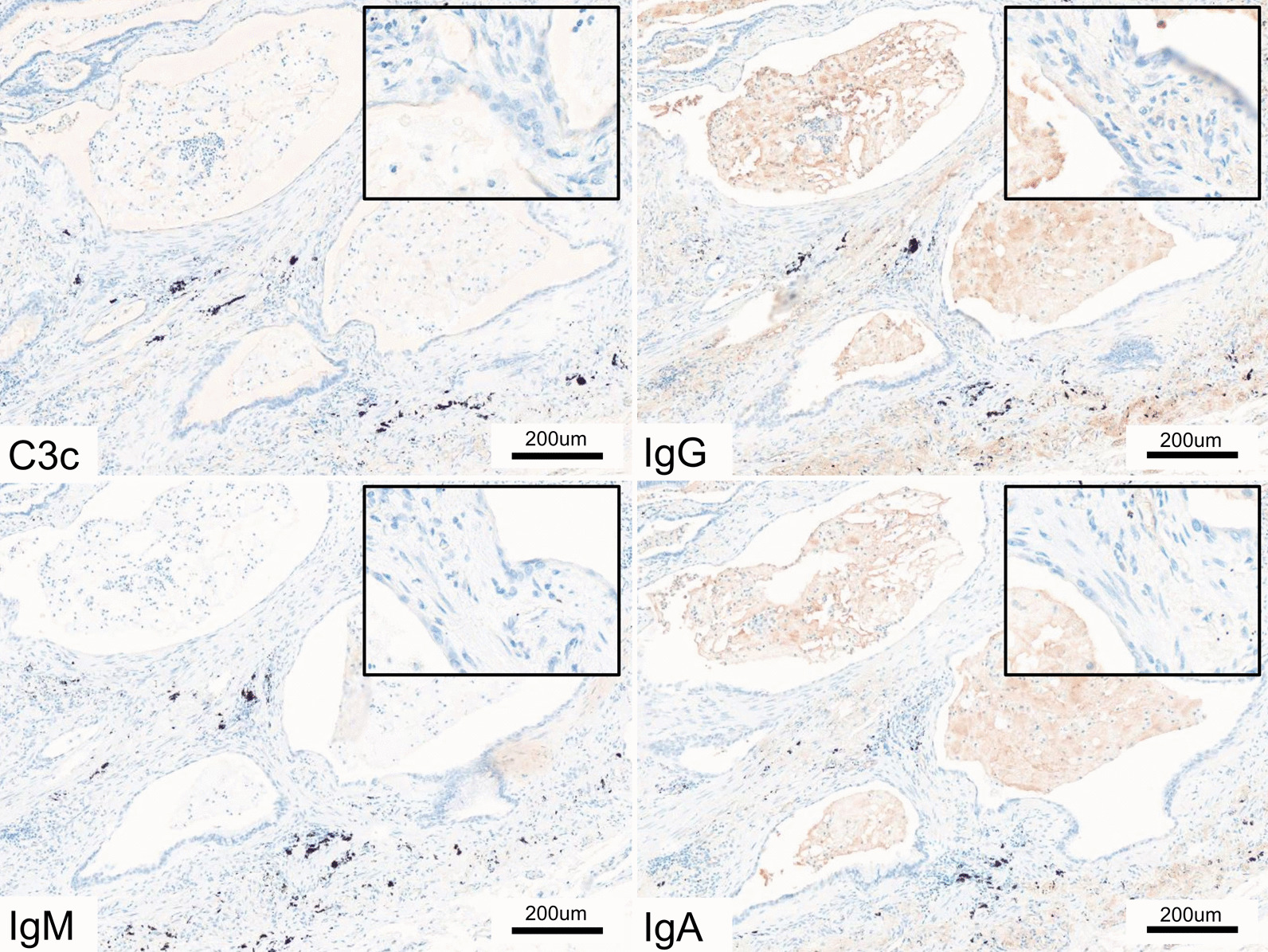
IHC staining with complement protein and Ig in the lungs of a patient with IPF. This patient was negative for complement C3c and Ig

Figure 4 shows the expression of complement C3c and Ig in the lungs of patients with IPF. Quantitative immunohistochemical analysis for IgG and IgA showed double-positive results in 15/19 IPF cases. IgG expression was weak (n = 10) or moderate (n = 5) in the lungs of 15 IPF cases. IgA expression was weak (n = 9) or moderate (n = 6) in the lungs of 15 IPF cases. Complement C3c and IgM were absent in 17/19 IPF cases (Table 2, Additional file 3: Fig. S3).

In comparison with the IPF group, the DM-ILD group showed a significantly higher expression of complement C3c (p < 0.001), IgG (p < 0.001), and IgM (p = 0.001) (Table 2, Figs. 3 and 4). Further, we performed separate statistical analyses for acute and chronic clinical courses (Table 3). We compared 9 DM-ILD patients who met the criteria for RP-ILD (acute course) and 9 IPF patients showing AEs; the DM-ILD group showed significantly higher expression of complement C3c (p < 0.001) and IgG (p = 0.011). Compared with IPF with a chronic clinical course (n = 10), DM-ILD with a chronic clinical course (n = 9) showed significantly higher expression of C3c (p = 0.011), IgG (p = 0.037) and IgM (p = 0.001).

**Table 3:** Subset analysis of immunohistochemical staining performed for clinical course

Expression intensity 0/1/2/3 (Mean ± SD)	Acute course^a^			Chronic course^b^		
	DM-ILD (n = 9)	IPF (n = 9)	P value	DM-ILD (n = 9)	IPF (n = 10)	P value
C3c	0/3/4/2 (1.889 ± 0.782)	8/1/0/0 (0.111 ± 0.333)	< 0.001	3/4/2/0 (0.889 ± 0.782)	9/1/0/0 (0.100 ± 0.316)	0.007
IgG	0/2/5/2 (2.000 ± 0.707)	3/4/2/0 (0.889 ± 0.782)	0.011	0/2/7/0 (1.778 ± 0.441)	1/6/3/0 (1.200 ± 0.632)	0.037
IgM	5/3/1/0 (0.556 ± 0.726)	7/2/0/0 (0.222 ± 0.441)	0.286	2/7/0/0 (0.778 ± 0.441)	10/0/0/0 (0.000 ± 0.000)	0.001
IgA	1/6/0/2 (1.333 ± 1.000)	3/2/4/0 (1.111 ± 0.928)	0.779	4/5/0/0 (0.556 ± 0.527)	1/7/2/0 (1.100 ± 0.568)	0.051
MDA5	0/0/8/1 (2.111 ± 0.333)	0/0/7/2 (2.222 ± 0.441)	0.539	0/1/7/1 (2.000 ± 0.500)	0/0/7/3 (2.300 ± 0.483)	0.203

Expression intensity score 0, negative staining; score 1, weakly positive; score 2, moderately positive; and score 3, strongly positive. Details are described in the “Methods” section

*DM* dermatomyositis; *ILD* interstitial lung disease; *IPF* idiopathic pulmonary fibrosis; *MDA5* melanoma differentiation-associated gene 5

^a^Acute course: rapidly progressive ILD (RP-ILD) in patients with DM-ILD (n = 9) vs. Acute exacerbation (AE) in patients with IPF (n = 9)

^b^Chronic course: non-RP-ILD in patients with DM-ILD (n = 9) vs. Non-AE in patients with IPF (n = 10)

Of the 18 patients (9 acute and 9 chronic courses) in the DM-ILD groups showed that the expression of C3c, and not IgG, IgM, IgA, or MDA5, was significantly (p = 0.022) higher in the acute course compared to that in the chronic courses. In contrast, in the 19 patients (9 acute and 10 chronic courses) in the IPF groups, there was no significant difference in the expression of C3c, IgG, IgM, IgA, and MDA5 in the acute course compared to the chronic courses. Additionally, there was no significant difference in the histological patterns in terms of the expression of C3c, IgA, IgG, IgM, or MDA5 between cases with DAD pattern (n = 11) and those with NSIP pattern (n = 6) in the DM-ILD group. Notably, the UIP pattern was only observed in one case (Table 1).

### Subset analysis: DM-ILD patients with and without anti-MDA5 antibody

Subsequently, we performed a subset analysis of DM-ILD patients who showed positive (n = 9) and negative (n = 9) results for anti-MDA5 antibodies (Table 4). In comparison with DM-ILD patients without anti-MDA5 antibodies, those showing anti-MDA5 antibody significantly (p < 0.001) showed a histological DAD pattern in tissue samples collected at autopsy (p = 0.002). Notably, in comparison with DM-ILD without anti-MDA5 antibody, cases with the anti-MDA5 antibody showed significantly higher C3c expression in the lungs (p = 0.015). However, no significant between-group difference was observed in the expression of IgG, IgA, IgM, and MDA5.

**Table 4:** Subset analysis of DM-ILD with and without anti-MDA5 antibody

	With MDA5 Ab	Without MDA5 Ab^b^	P value
Number	9	9	
Age	55 (48–66)	53 (44–63)	0.659
Sex: male	5 (56%)	1 (11%)	0.131
Clinical course: RP-ILD	7 (78%)	2 (22%)	0.057
Tissue collection method			0.002
SLB	3 (33%)	8 (89%)	
Transplant	0 (0%)	1 (11%)	
Autopsy	6 (67%)	0 (0%)	
Dx of IIMs			0.620
DM	5 (56%)	7 (78%)	
CADM	4 (44%)	2 (22%)	
Histological pattern			< 0.001
DAD	9 (100%)	2 (22%)	
NSIP	0 (0%)	6 (67%)	
UIP	0 (0%)	1 (11%)	
IHC staining^a^			
C3c	0/3/4/2 (1.889 ± 0.782)	3/4/2/0 (0.889 ± 0.782)	0.022
IgG	0/2/5/2 (2.000 ± 0.707)	0/2/7/0 (1.778 ± 0.441)	0.458
IgA	1/6/0/2 (1.333 ± 1.000)	4/5/0/0 (0.556 ± 0.527)	0.060
IgM	5/3/1/0 (0.556 ± 0.726)	2/7/0/0 (0.778 ± 0.441)	0.315
MDA5	0/0/8/1 (2.111 ± 0.333)	0/1/7/1 (2.000 ± 0.500)	0.586

*CADM* clinical amyopathic dermatomyositis; *DAD* diffuse alveolar damage; DM dermatomyositis; *Dx* diagnosis; *IHC* immunohistochemistry; *IIMs* idiopathic inflammatory myopathies; *ILD* interstitial lung disease; *MDA5* melanoma differentiation-associated gene 5; *NSIP* nonspecific interstitial pneumonia; *RP-ILD* rapidly progressive interstitial lung disease; *SLB* surgical lung biopsy; *UIP* usual interstitial pneumonia

Expression intensity score 0, negative staining; score 1, weakly positive; score 2, moderately positive; and score 3, strongly positive. Details were described in the “Methods” section

^a^IHC staining items are indicated by intensity scores of 0/1/2/3 and mean ± SD

^b^Patients without anti-MDA5 antibody were all positive for anti-Aminoacyl-tRNA Synthetase antibodies

### Lung injury in human MDA5 transgenic mice

**Fig. 5:**
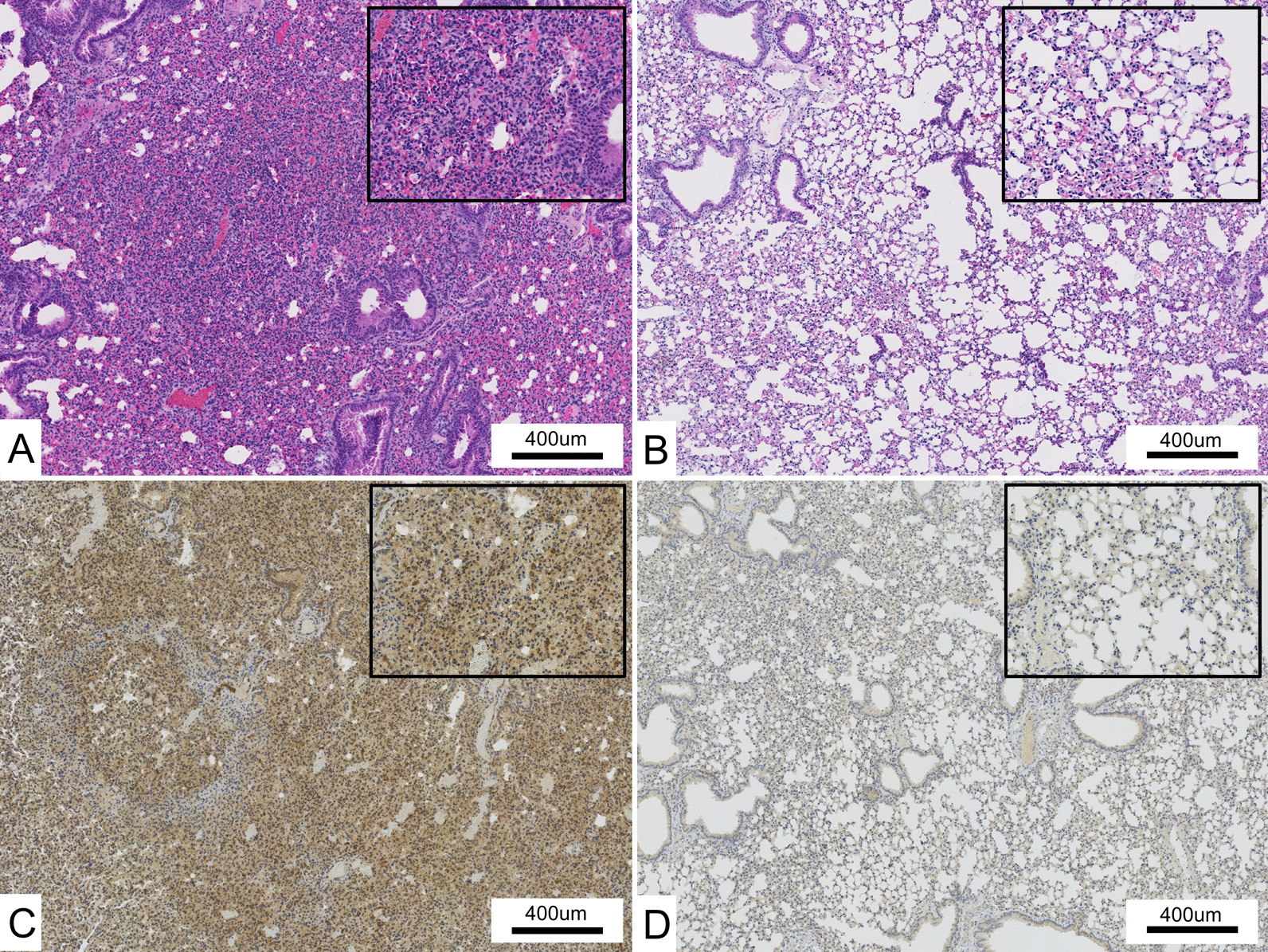
Histopathological findings in the lungs of lung injury model mouse. **A** H&E staining of lung injury model mouse (transgenic mice treated with antisera) showing severe inflammatory cells infiltration with alveolar collapse. **B** H&E staining of control mouse (transgenic mice treated with rabbit sera). This shows only very mild inflammatory cells infiltration. **C** IHC staining with anti-human MDA5 mAb (clone H27) in lung injury model mouse showing extensive and strong expression in the alveolar epithelium. **D** IHC staining with anti-MDA5 mAb (clone H27) in control mouse showing very low expression in the alveolar epithelium

We generated three lines (lines no. 32, 55, 116) of human MDA5 transgenic mice with overexpression of human MDA5 proteins in the lungs and treated them with anti-MDA5 polyclonal antibodies (antisera) to create a new lung injury model. We also administered normal rabbit sera to transgenic mice and used them as control mice and administered antisera or normal rabbit sera to wild-type (BDF1) mice. Treatment with antisera or rabbit sera did not induce severe lung injury in wild-type BDF1 mice. In contrast, treatment with an anti-MDA5 polyclonal antibodies induced lung injury in all three lines of MDA5 transgenic mice. In the transgenic mice treated with antisera, the alveolar septum was strongly infiltrated with lymphocytes, and many of the alveoli had collapsed (Fig. 5a). Transgenic mice treated with rabbit sera also showed inflammatory cell infiltration of the alveolar septum, but it was more reduced than that observed in the lung injury model (Fig. 5b). Transgenic mice treated with purified rabbit anti-MDA5 polyclonal antibodies also induced lung injury and showed inflammatory cell infiltration of the alveolar septum. In contrast, transgenic mice treated with rabbit IgG did not induce lung injury (data not shown).

### Human-MDA5 expression in human MDA5 transgenic mice

In the lungs of human MDA5 transgenic mice treated with anti-MDA5 antibodies (lung injury model mouse), human MDA5 protein was strongly expressed in alveolar epithelium and macrophages in the alveoli (Fig. 5c, h, e; staining in Fig. 5a). Very low human MDA5 protein expression was also observed in transgenic mice treated with rabbit sera (control mouse) (Fig. 5d, h, e; staining in Fig. 5b). The kidneys of the lung injury model mice showed glomerular atrophy and lymphocytic infiltration. However, control mice rarely showed glomerular atrophy and lymphocytic infiltration (Additional file 4: Fig. S4a, b). Human MDA5 protein was strongly expressed in the renal tubular epithelial cells and glomeruli in lung injury model mice, while control mice showed very weak MDA5 expression in renal tubular epithelial cells and negative expression in the glomeruli (Additional file 4: Fig. S4c, d).

### Strong expression of complement and Ig in human MDA5 transgenic mice

**Fig. 6:**
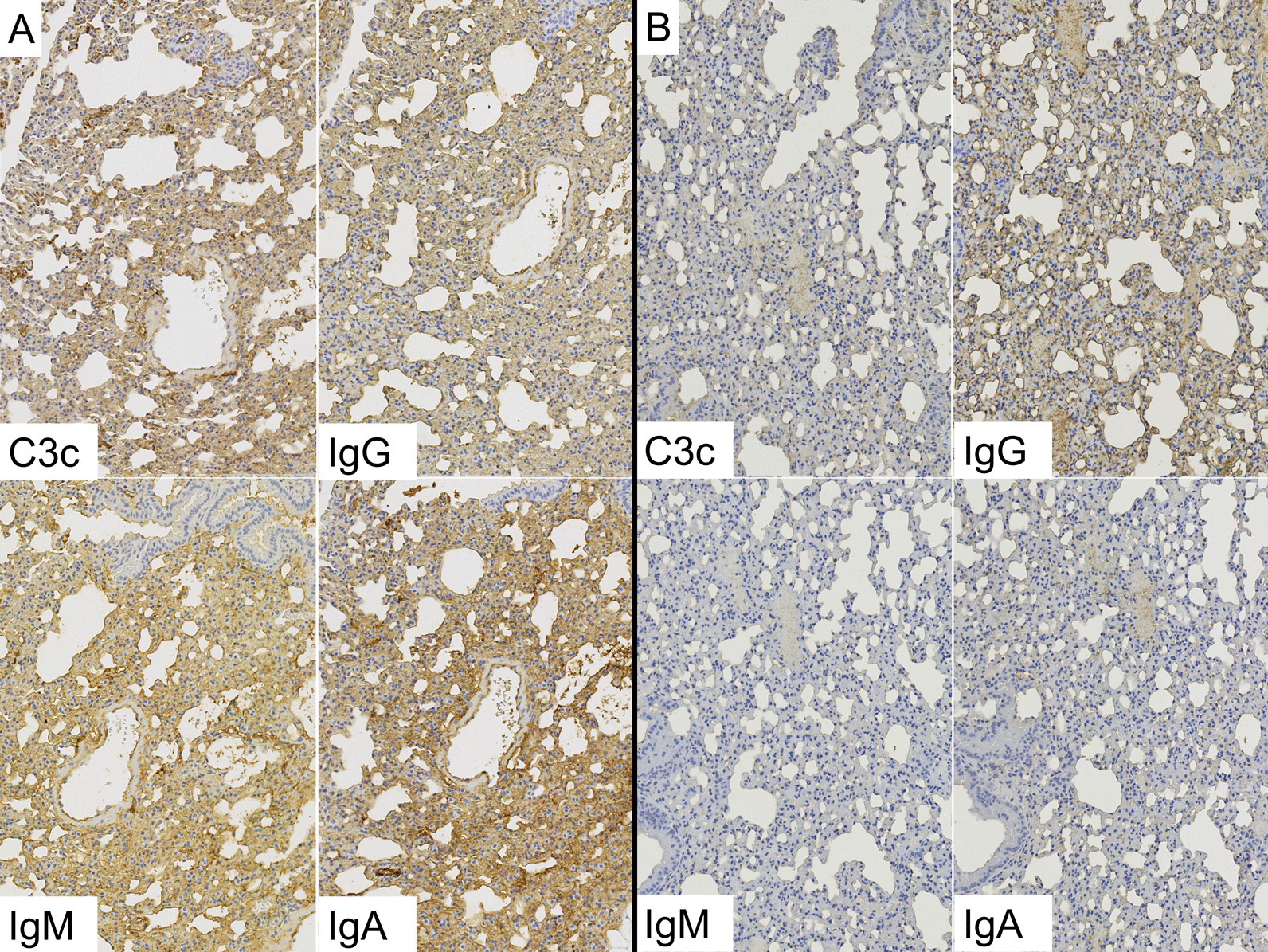
Immunohistochemical (IHC) analysis for complement protein and Ig in the lungs of the lung injury model mice. **A** Lung injury model mice (transgenic mice treated with antisera) showed moderate to severe expression of C3 and Ig. **B** Control mice (transgenic mice treated with rabbit sera) showed moderate expression of IgG but showed very mild expression of the other immunoglobulins and C3

IgG, IgM, IgA, and complement (C3) were all strongly expressed in the lungs of the lung injury model. IgG, IgM, IgA, and C3 were strongly expressed in the alveolar epithelium, where human MDA5 was strongly expressed (Fig. 6). When the human MDA5 lung injury models at 4 and 8 weeks were compared, slightly stronger Ig and C3 expression was observed in the mice grown for 8 weeks (Additional file 5: Fig. S5a, b). Although human MDA5 transgenic mice injected with normal rabbit serum also expressed complement and Ig, the expression was less pronounced than that seen in the lung injury model and stronger than that seen in wild-type mice treated with normal rabbit sera (Additional file 5: Fig. S5c, d). In the kidney of the lung injury model, IgG, IgM, IgA, and C3 were all strongly expressed in the glomeruli, where human MDA5 was strongly expressed, in comparison with the corresponding expression levels in the control mice (Additional file 6: Fig. S6).

## Discussion

This study showed that C3c, IgG, and IgM, but not IgA, were more strongly expressed in the injured alveolar epithelium of patients with DM-ILD than in patients with IPF (used as control patients), suggesting that C3c, IgG, and IgM are crucially involved in DM-ILD-related lung injury. In the present study, no difference in MDA5 expression was observed between DM-ILD and IPF, indicating that the intensity of MDA5 expression in lung tissue is not involved in the development of DM-ILD. On the other hand, complement and immunoglobulins were expressed much more strongly in DM-ILD than in IPF, suggestive of the formation of immune complexes. Immune complex deposition, which is the pathogenesis of type III hypersensitivity, is a prominent characteristic of several lung-damaging autoimmune diseases, including hypersensitivity pneumonitis (e.g., farmers’ lungs, summer-type pneumonitis), systemic lupus erythematosus, cryoglobulinemia, rheumatoid arthritis, scleroderma, and Sjögren’s syndrome [14–16]. Thus, while type III hypersensitivity reactions can cause lung damage in patients with DM-ILD, the contribution of type III hypersensitivity reactions to lung injury in DM remains unclear. This is the first report outlining the relationship between DM-ILD and type III hypersensitivity reactions.

We also established human MDA5 transgenic mice and anti-human MDA5 antibodies in this study. By administering anti-human MDA5 polyclonal antibody to human MDA5 transgenic mice, we created a new mouse model of autoantibody-induced lung injury. This mouse model showed strong expression of complement and Ig in the alveolar epithelium, as in human specimens. Our in vivo model proved the involvement of type III hypersensitivity reactions in the lung injury associated with DM-ILD.

Funabiki et al. [26] reported that a mutant mouse line harboring a single missense mutation G821S in MDA5 spontaneously developed lupus-like nephritis and systemic autoimmune symptoms. These mice presented with chronic inflammation and nephritis, showing Ig and complement deposition, upregulation of inflammatory cytokines and chemokines in the kidney, increased Ig expression, and serum positivity for anti-nuclear and anti-DNA antibodies. Similar observations were obtained in our lung injury model mouse. Taken together, chronic inflammation can occur in various organs, including the lungs, which may explain the chronic course and fibrotic changes in the lungs of some DM-ILD patients.

Myositis-specific antibodies are relevant for the diagnosis and prognosis prediction of IIMs. Several myositis-specific antibodies have been recently reported in patients with IIMs, including transcription intermediary factor 1 gamma (TIF1γ), Mi-2, small ubiquitin-like modifier activating enzyme (SAE), aminoacyl-transfer (t)RNA synthetases (ARS) including anti-Jo-1, and MDA5 [6]. This study showed lung deposition of immune complexes consisting of Ig and complement C3 in patients with DM who showed positive or negative findings for the anti-MDA5 antibody, indicating deposition of immune complexes in patients with DM who are positive for other myositis-specific antibodies for TIF1γ, Mi-2, SAE, and ARS. Further analysis is required to verify this finding.

Some recent studies have shown the effectiveness of plasma-exchange therapy in DM-ILD patients showing RP-ILD [27, 28]. Abe et al. reported that 6/10 DM-ILD patients showing RP-ILD who were treated with plasma-exchange therapy, as well as corticosteroids and calcineurin inhibitors, had a better prognosis than those who did not undergo plasma-exchange therapy [27]. Moreover, plasma-exchange therapy is a treatment option for SLE caused by type III hypersensitivity reactions that cannot be controlled with corticosteroids or other immunosuppressive therapies [29]. In cases of DM-ILD caused by a type III hypersensitivity reaction, as shown in this study, plasma-exchange therapy could effectively remove complement components, Ig, and inflammatory cytokines. Plasma-exchange therapy could be an alternative therapeutic option for DM-ILD patients showing RP-ILD. A future prospective large-scale clinical study should clarify this hypothesis.

This study had two limitations. First, this was a small cross-sectional study; moreover, tissues were collected using a combination of SLB and autopsy. Thus, histological changes in the agonal stage in autopsy cases could have resulted in a bias in the evaluation. Second, only IHC staining was used to examine the expression of complement proteins and Ig. There was some unreliability in the evaluation of IHC staining, although the staining method was fixed, and the slides were evaluated by two pathologists who were blinded to the study. Future studies, utilizing additional approaches in the analyses will be needed to confirm our hypotheses.

## Supplementary Information

**Additional file 1: Figure S1.** Semi-quantitative immunohistochemical (IHC) scoring of C3c. We evaluated the staining intensity in the alveolar epithelium over four levels: score 0, negative; score 1, weakly positive; score 2, moderately positive; and score 3, strongly positive.

**Additional file 2: Figure S2.** Semi-quantitative immunohistochemical (IHC) scoring of IgG, IgM, and IgA expression. These images were obtained after IgG staining; IgA and IgM were evaluated similar to the process shown in the pictures.

**Additional file 3: Figure S3.** Results of statistical analysis for scoring of C3c, IgG, IgM, and IgA expression.

**Additional file 4: Figure S4.** Histopathological findings in the kidney of the mouse model. (**A**) H&E staining of the lung injury model mice (transgenic mice treated with antisera) showing lymphocyte infiltration and atrophy of the glomerulus. (**B**) H&E staining of the control mice (transgenic mice treated with rabbit sera) showing no abnormalities. (**C**) IHC staining with anti-MDA5 mAb (clone H27) in the lung injury model mice showing moderate positivity in renal tubular epithelial cells and strongly positivity in glomeruli. (**D**) IHC staining with the anti-MDA5 mAb (clone H27) in control mice showing weak expression in renal tubular epithelial cells but no expression in glomeruli.

**Additional file 5: Figure S5.** Expression of C3 and IgG in several mouse models. (**A**) The lung injury model grown for 4 weeks. (**B**) The lung injury model grown for 8 weeks. (**C**) Human MDA5 transgenic mice treated with control rabbit serum. (**D**) Wild-type mouse treated with control rabbit serum.

**Additional file 6: Figure S6.** IHC staining with complement protein and Ig in the kidney of mouse model. (**A**) The lung injury model showed severe expression of C3 and Ig in the glomeruli. (**B**) The control mouse showed almost no C3 and Ig expression.

## Data Availability

The datasets used and/or analyzed during the current study available from the corresponding author on reasonable request.
