# Supplementary figures and images for: Enhanced immune complex formation in the lungs of patients with dermatomyositis
Source: Respir Res. 2023 Mar 19;24:86. doi: 10.1186/s12931-023-02362-0 (PMC10024827; doi:10.1186/s12931-023-02362-0)

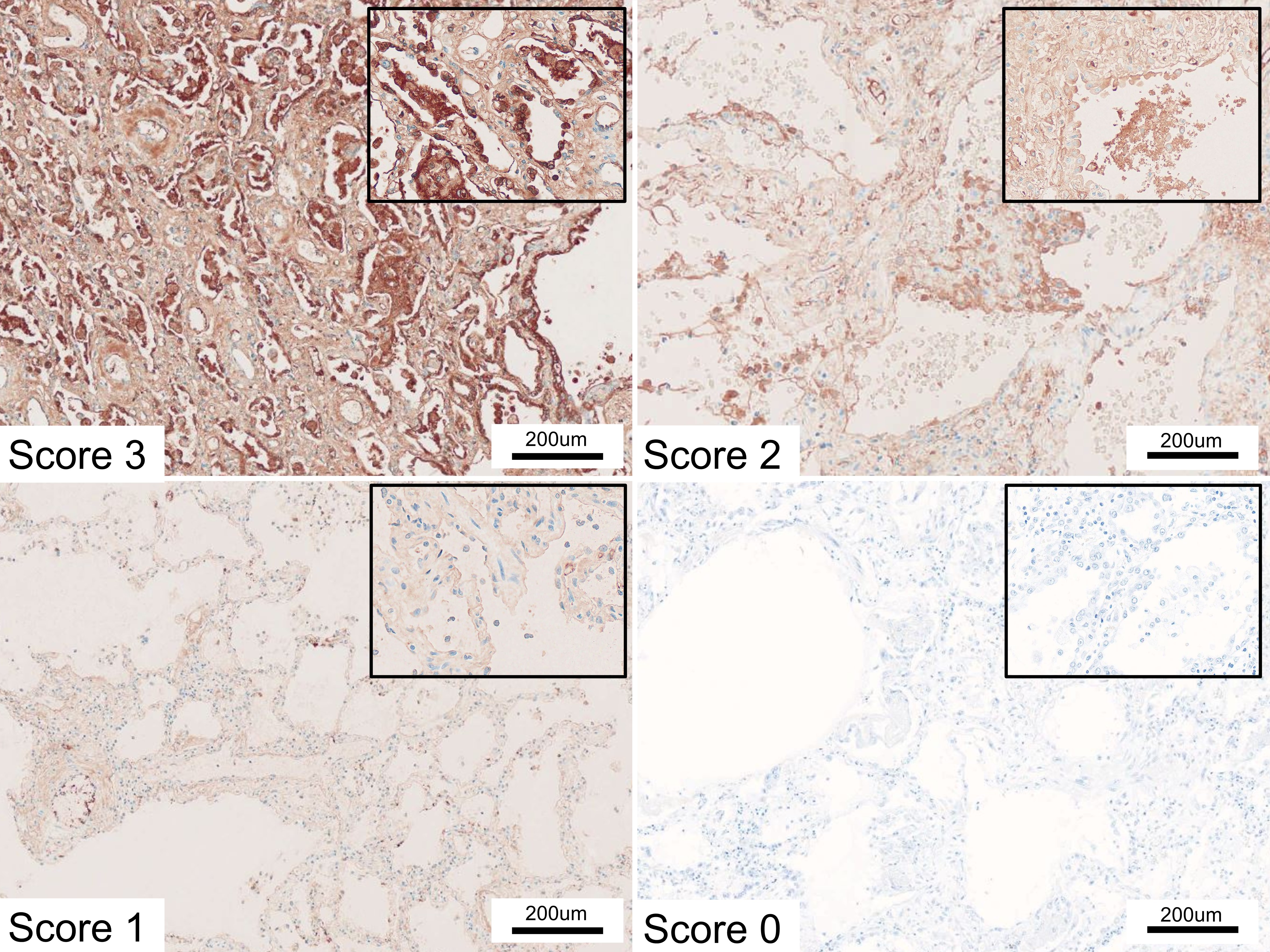

Supplement: Supplementary file 1 — Additional file 1: Figure S1. Semi-quantitative immunohistochemical (IHC) scoring of C3c. We evaluated the staining intensity in the alveolar epithelium over four levels: score 0, negative; score 1, weakly positive; score 2, moderately positive; and score 3, strongly positive. [file 12931_2023_2362_MOESM1_ESM.jpg]

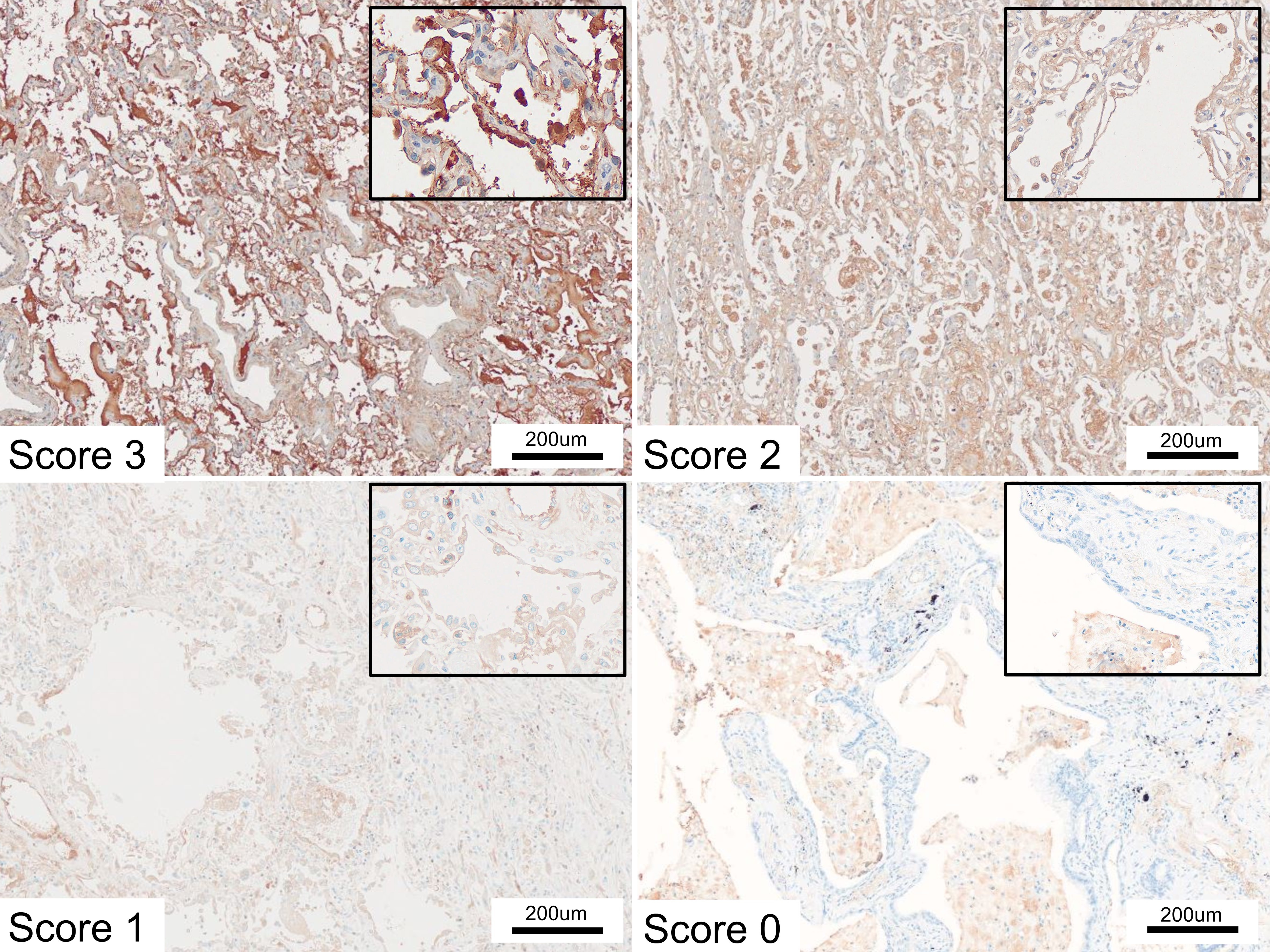

Supplement: Supplementary file 2 — Additional file 2: Figure S2. Semi-quantitative immunohistochemical (IHC) scoring of IgG, IgM, and IgA expression. These images were obtained after IgG staining; IgA and IgM were evaluated similar to the process shown in the pictures. [file 12931_2023_2362_MOESM2_ESM.jpg]

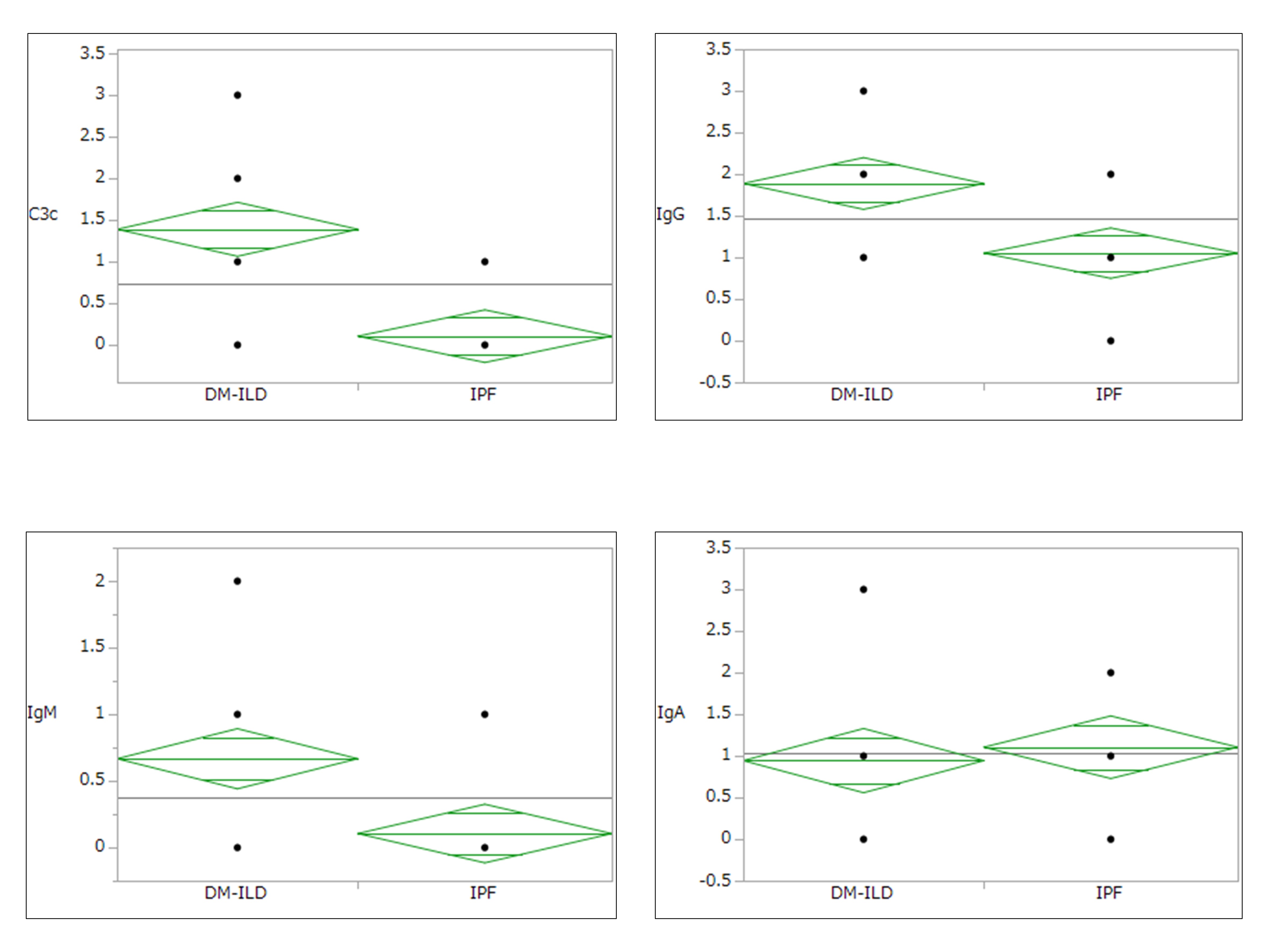

Supplement: Supplementary file 3 — Additional file 3: Figure S3. Results of statistical analysis for scoring of C3c, IgG, IgM, and IgA expression. [file 12931_2023_2362_MOESM3_ESM.jpg]

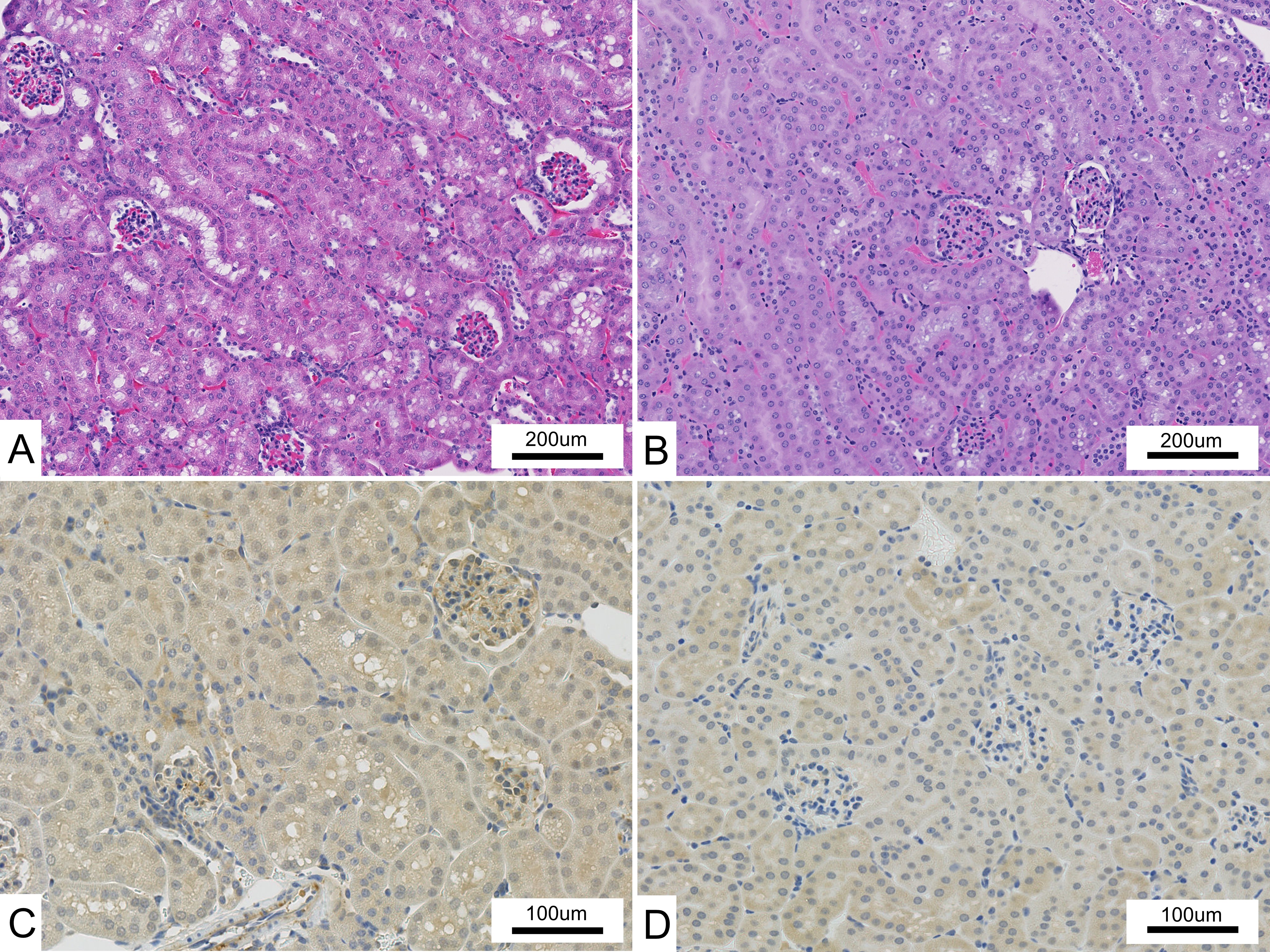

Supplement: Supplementary file 4 — Additional file 4: Figure S4. Histopathological findings in the kidney of the mouse model. (A) H&E staining of the lung injury model mice (transgenic mice treated with antisera) showing lymphocyte infiltration and atrophy of the glomerulus. (B) H&E staining of the control mice (transgenic mice treated with rabbit sera) showing no abnormalities. (C) IHC staining with anti-MDA5 mAb (clone H27) in the lung injury model mice showing moderate positivity in renal tubular epithelial cells and strongly positivity in glomeruli. (D) IHC staining with the anti-MDA5 mAb (clone H27) in control mice showing weak expression in renal tubular epithelial cells but no expression in glomeruli. [file 12931_2023_2362_MOESM4_ESM.jpg]

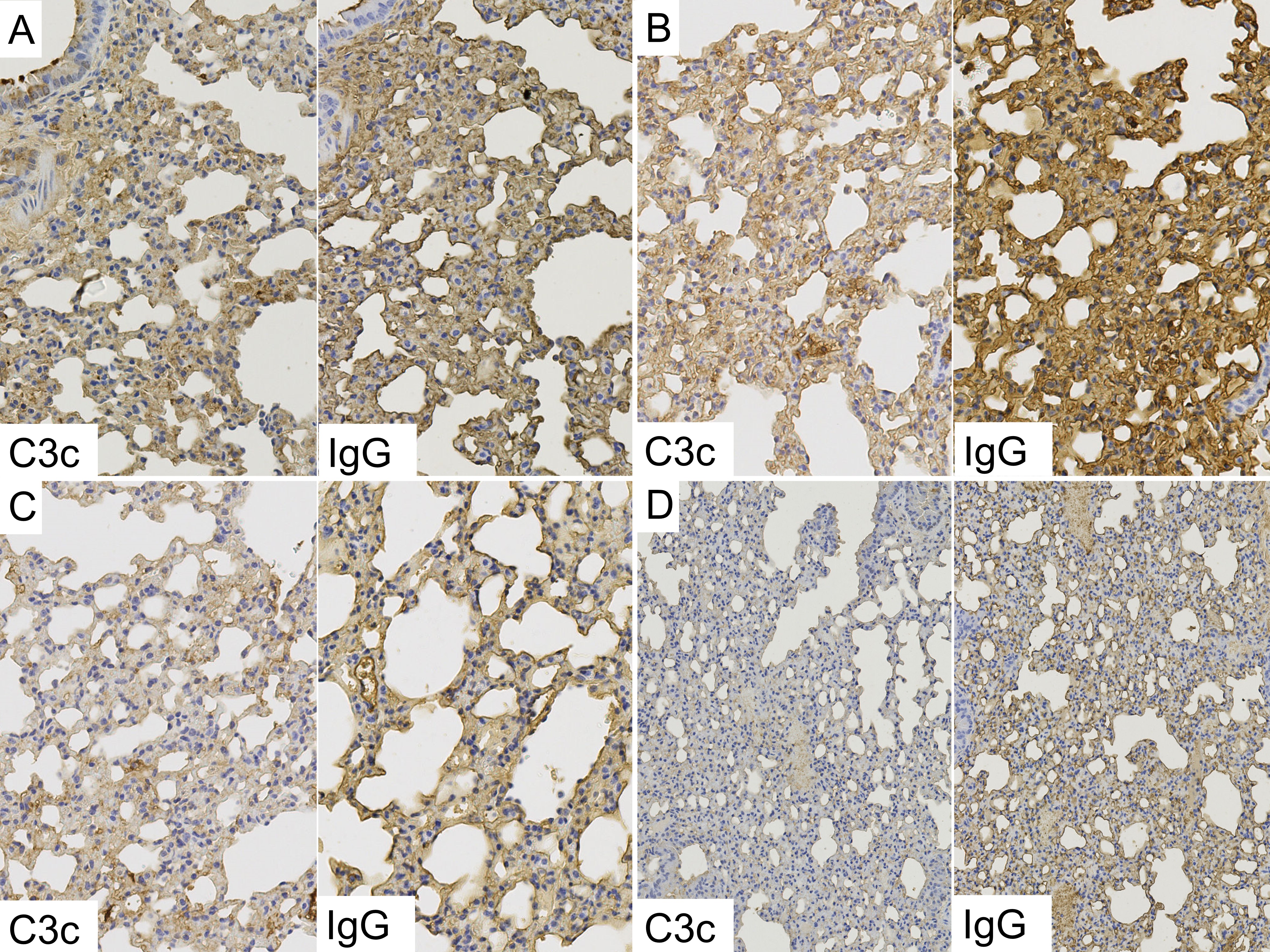

Supplement: Supplementary file 5 — Additional file 5: Figure S5. Expression of C3 and IgG in several mouse models. (A) The lung injury model grown for 4 weeks. (B) The lung injury model grown for 8 weeks. (C) Human MDA5 transgenic mice treated with control rabbit serum. (D) Wild-type mouse treated with control rabbit serum. [file 12931_2023_2362_MOESM5_ESM.jpg]

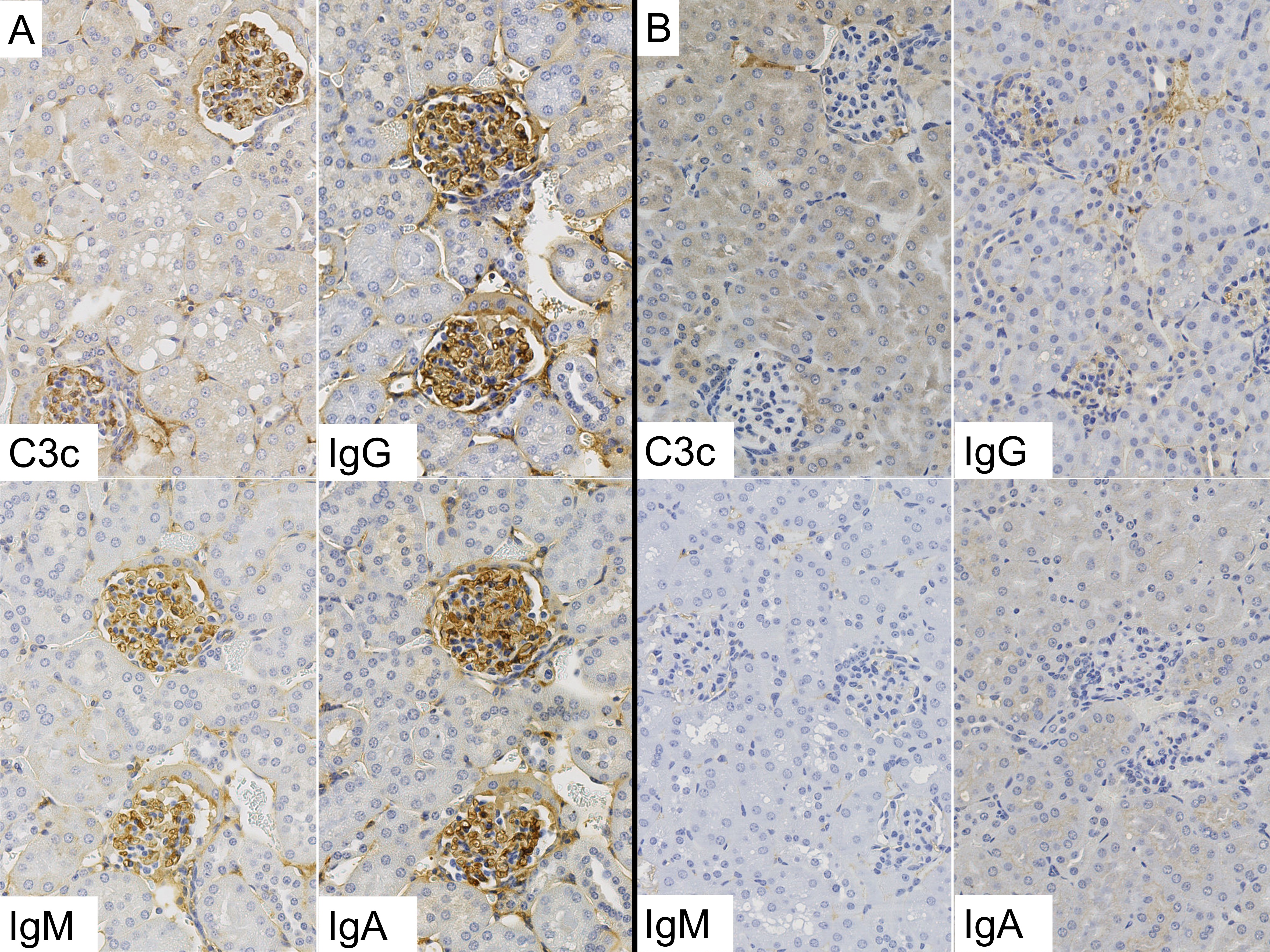

Supplement: Supplementary file 6 — Additional file 6: Figure S6. IHC staining with complement protein and Ig in the kidney of mouse model. (A) The lung injury model showed severe expression of C3 and Ig in the glomeruli. (B) The control mouse showed almost no C3 and Ig expression. [file 12931_2023_2362_MOESM6_ESM.jpg]
